# Ultrasonographic and pathological findings of pseudomyogenic hemangioendothelioma

**DOI:** 10.1002/kjm2.12828

**Published:** 2024-04-09

**Authors:** Gui‐Wu Chen, Xiao‐Ling Leng, Shao‐Ming Liu, Xiao‐Min Liao

**Affiliations:** ^1^ Department of Ultrasound The Tenth Affiliated Hospital of Southern Medical University, Dongguan People's Hospital Dongguan Guangdong China; ^2^ Department of Pathology The Tenth Affiliated Hospital of Southern Medical University, Dongguan People's Hospital Dongguan Guangdong China

Pseudomyogenic hemangioendothelioma (PHE) is an indolent and low‐grade tumor that often mimics other benign and malignant lesions, making accurate diagnosis crucial for effective patient management.^1^ The variable clinical presentations and pathological features of PHE pose diagnostic challenges for inexperienced radiologists.^2^ Here, we present a 17‐year‐old man with PHE, characterized by ultrasonography and magnetic resonance imaging, and confirmed by pathological examination.

A 17‐year‐old male presented to our hospital with a complaint of pain in his leg while walking for the past 10 months. During the physical examination, multiple palpable masses were detected in the right thigh, which were hard, ill‐defined, and had poor mobility. High‐frequency ultrasound revealed that the largest mass in the muscle of the right thigh was hypoechoic, well‐defined, and irregular (Figure 1A), with a few blood flow signals both inside and around the mass (Figure 1B). Magnetic resonance imaging suggested that the largest mass appeared as a high signal on T1‐weighted (Figure 1C) and T2‐weighted imaging (Figure 1D), with significant enhancement (Figure 1E). Finally, the patient underwent a surgical resection of masses, and a pathological examination confirmed the diagnosis of PHE. Hematoxylin and eosin staining revealed a diffuse growth of epithelioid cells with abundant cytoplasm and slightly off‐centered nuclei (Figure 1F). Immunohistochemical staining results were positive for ERG, Fli‐1, INI‐1, Vim, and partially positive for CD31. Weakly positive staining was observed for CK, SMA, Cal, desmon, while CD34, CD56, Desmin, MyoD1, Myogenin, S‐100, WT‐1, EMA, HMB‐45, Melan‐A were negative. The Ki‐67 proliferation index was approximately 5%.

**FIGURE 1:**
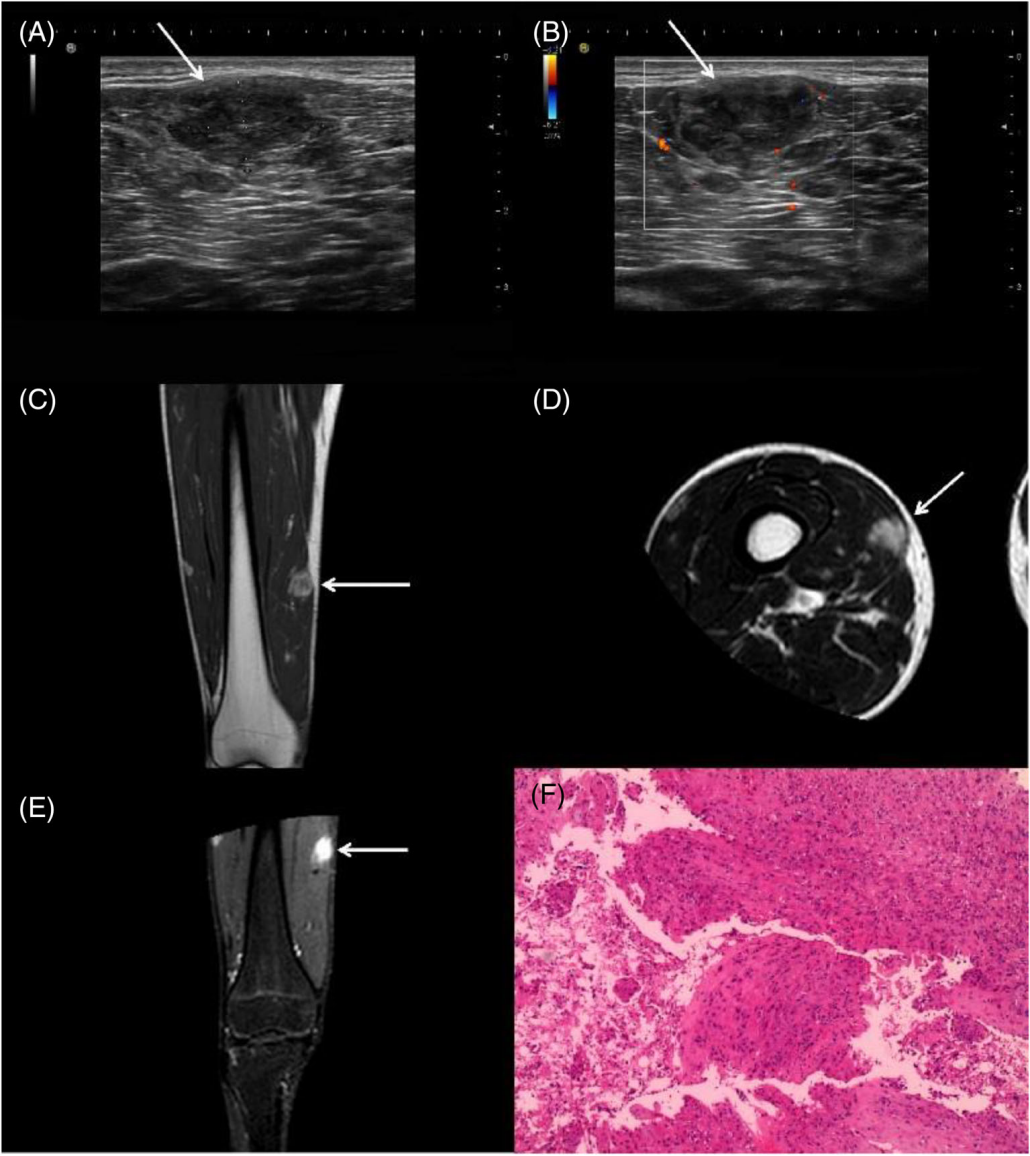
(A) Grayscale ultrasound showed the largest mass (arrow) was hypoechoic, well‐defined, and irregular. (B) Color Doppler flow imaging showed a few blood flow signals both within and around the largest mass (arrow). (C) T1‐weighted imaging showed the largest mass (arrow) appeared as a high‐intensity signal. (D) T2‐weighted imaging showed the largest mass (arrow) appeared as a high‐intensity signal. (E) Enhanced magnetic resonance imaging showed the largest mass (arrow) with significant enhancement. (F) Hematoxylin and eosin staining showed a diffuse growth of epithelioid cells with abundant cytoplasm, slightly off‐centered nuclei, distinct nucleoli, and infrequent mitotic figures.

PHE is a rare vascular tumor that mainly affects young patients, with a male predominance, and can present in various locations throughout the body, including the head, esophagus, neck, chest wall, breast, trunk, limbs, pelvis, and external genitalia. It can involve multiple tissue planes, including the dermis, subcutaneous tissue, bone, and skeletal muscle.^3^ Given its variable clinical presentations and pathological features, inexperienced radiologists may be prone to misdiagnosing PHE as dermatofibroma, epithelioid sarcoma, rhabdomyosarcoma, or other similar lesions.^4^ In our case, high‐frequency ultrasound showed PHE was hypoechoic, well‐defined, and irregular with a few blood flow signals both inside and around the mass while magnetic resonance imaging showed a high signal with significant enhancement. Accurate diagnosis is crucial for effective patient management and requires expertise in both clinical and pathological domains. Therefore, it is essential to consider PHE in the differential diagnosis of any soft‐tissue mass with variable clinical and radiological presentations.

## CONFLICT OF INTEREST STATEMENT

The authors declare no conflict of interest.

## CONSENT FOR PUBLICATION

Written informed consent was obtained from the patient to publish this manuscript in accordance with the journal's patient consent policy.
